# Fruit Characteristics, Peel Nutritional Compositions, and Their Relationships with Mango Peel Pectin Quality

**DOI:** 10.3390/plants10061148

**Published:** 2021-06-04

**Authors:** Malaiporn Wongkaew, Sila Kittiwachana, Nutthatida Phuangsaijai, Bow Tinpovong, Chantalak Tiyayon, Tonapha Pusadee, Bajaree Chuttong, Korawan Sringarm, Farhan M. Bhat, Sarana Rose Sommano, Ratchadawan Cheewangkoon

**Affiliations:** 1 Interdisciplinary Program in Biotechnology, Graduate School, Chiang Mai University, Chiang Mai 50200, Thailand; malaiporn_wongkaew@cmu.ac.th; 2 Program of Food Production and Innovation, Faculty of Integrated Science and Technology, Rajamangala University of Technology Lanna, Chiang Mai 50300, Thailand; bowtinpovong@rmutl.ac.th; 3 Plant Bioactive Compound Laboratory, Faculty of Agriculture, Chiang Mai University, Chiang Mai 50200, Thailand; 4 Department of Chemistry, Faculty of Science, Chiang Mai University, Chiang Mai 50200, Thailand; silacmu@gmail.com (S.K.); nutthatida_phu@cmu.ac.th (N.P.); 5 Department of Plant and Soil Sciences, Faculty of Agriculture, Chiang Mai University, Chiang Mai 50200, Thailand; chantalak.t@cmu.ac.th (C.T.); tonapha.p@cmu.ac.th (T.P.); 6 Department of Entomology and Plant Pathology, Faculty of Agriculture, Chiang Mai University, Chiang Mai 50200, Thailand; bajaree.c@cmu.ac.th; 7 Innovative Agriculture Research Center, Faculty of Agriculture, Chiang Mai University, Chiang Mai 50200, Thailand; 8 Department of Animal and Aquatic Science, Facuslty of Agriculture, Chiang Mai University, Chiang Mai 50200, Thailand; korawan.s@cmu.ac.th; 9 Department of Food Engineering and Technology, Sant Longowal Institute of Engineering and Technology, Longowal, Punjab 148106, India; farhanbhat999@gmail.com

**Keywords:** biomass valorization, fruit physiology, fruit peel pectin, microwave-assisted extraction, partial least-squares regression

## Abstract

Mango peel, a byproduct from the mango processing industry, is a potential source of food-grade mango peel pectin (MPP). Nonetheless, the influence of fruit physical characteristics and phytochemicals of peels on their correspondent pectin level has never been examined, particularly when high-quality food additives are of commercial need. Subsequently, the ultimate aim of the present study was to comprehend their relationship using chemometric data analyses as part of raw material sourcing criteria. Principal component analysis (PCA) advised that mangoes of ‘mahachanok’ and ‘nam dok mai’ could be distinguished from ‘chok anan’ and ‘kaew’ on the basis of physiology, peel morphology, and phytochemical characteristics. Only pectin extracted from mango var. ‘chok anan’ was classified as low-methoxyl type (Mox value ~4%). Using the partial least-squares (PLS) regression, the multivariate correlation between the fruit and peel properties and the degree of esterification (DE) value was reported at *R*^2^ > 0.9 and *Q*^2^ > 0.8. The coefficient factors illustrated that yields of byproducts such as seed and total biomass negatively influenced DE values, while they were positively correlated with crude fiber and xylose contents of the peels. Overall, it is interesting to highlight that, regardless of the differences in fruit varieties, the amount of biomass and peel proximate properties can be proficiently applied to establish classification of desirable properties of the industrial MPP.

## 1. Introduction

Mango, the king of fruits with high nutritive value, is extensively cultivated in the tropical and subtropical regions [1]. It is one of the most important commercial fruit crops worldwide in terms of production, processing, and consumption [2,3]. In Thailand, approximately 300,000 tons of local varieties such as ‘mahachanok’, ‘chok anan’, ‘nam dok mai’, and ‘kaew’ mangoes, especially those of ripe fruits, are used for food processing [4]. The commercially processed products include preserved canned fruits, frozen slices, purée, juices, nectar, and various dehydrated products [5,6]. During processing, it is estimated that almost 200,000 tons of food loss is generated, and mango peels account for as much as 24% of those volumes [7]. More importantly, poor management of this industrial loss could have a great impact on the environment; therefore, attempts have been made in order to add value to these byproducts such as biomass from mango processing [8,9,10,11].

Mango peel is a potential source of dietary fiber, and it contains 5–11% pectin depending on fruit varieties and the extraction methods [12,13,14,15]. Additionally, it comprises various classes of polyphenols, carotenoids, and vitamins with excellent antioxidative and functional properties [16,17]. Therefore, this byproduct is a promising target for commercial valorization [18,19]. Previous reports indicated that the peel contains high contents of carbohydrates (80%), crude fiber (8%), and pectin (13%), as well as reasonable quantities of proteins (4%) and fats (2%) [20,21].

Pectin is a structural heteropolysaccharide found in the primary cell walls that provides mechanical strength and flexibility via interaction with other cell-wall components [22]. High contents of pectin can be found in almost all parts of fruits depending on the varieties and maturity stages [23,24,25,26,27]. The major constituent of pectin is poly (1,4)-α-d-galacturonan as a backbone with the carboxyl groups presenting in either free acid or methyl ester forms [28]. Pectin can be categorized into two classes according to the proportion of the esterified groups into low-methoxyl pectin (LMP) (DE < 50%) and high-methoxyl pectin (HMP) (DE > 50%). The latter is an excellent emulsifier and stabilizer which can be used as a gelling agent and thickening agent [29,30,31]. It is also used as a fat replacer and health-promoting functional food ingredient [10,32]. Additionally, pectin can be added to pharmaceutical products such as bioactive components, drug and gene delivery compounds, tissue engineering products, and wound healing patches [33].

To recover pectin from plant resources, microwave-assisted extraction (MAE) is more effective for the extraction of high-quality pectin than conventional heating [34,35,36,37]. Such a technique has been implemented in the recovery of pectin from dietary-rich biomasses such as banana peels [38] and orange peels [39], and it has shown greater success when applied to mango peels [11,12,40]. It is believed that the quality of raw materials is foremost responsible for the extractable quality of the pectin. Nevertheless, the relationship between the physicochemical properties of fruit and peel along and the chemical qualities of the pectin has never been reported, especially to develop an index for raw material sourcing. With this rationale, the objectives of the present study were first to evaluate the physiological and physicochemical characteristics along with the proximate values of mango peels from commercially available mango varieties. Then, their relationships with the chemical qualities of the MAE-extracted mango peel pectin (MPP) were evaluated. The research outcomes will be beneficial for setting up the selection criteria of the biological materials for MPP production on a substantial scale.

## 2. Results and Discussion

### 2.1. Physical Characteristics of Mango Fruit Varieties

The physical characteristics of the mango fruits are illustrated in Table 1, including color (L*, a*, b*), arithmetic mean diameter (D_a_), geometric mean diameter (D_g_), aspect ratio (R_a_), sphericity (Φ), surface area (S), peel-to-fruit ratio, and the percentage of peel, flesh, and seed.

Lightness (L*), redness (a*), and yellowness (b*) are considered the most informative parameters for quality assessment of agricultural produce due to its uniform distribution and close relationship with sensorial perception by humans [41,42]. The CIE color space has been used to determine maturity index and the ripening process of mangoes [43,44]. According to the results, L* values varied among different varieties ‘mahachanok’ (68.83), ‘chok anan’ (69.98), and ‘kaew’ (67.68), while ‘nam dok mai’ showed the highest luminosity (72.26). Lightness alteration of the fruit skin has been linked to an advancement of the ripening stage and deterioration of mango fruits [45,46,47]. The a* value is also a good indicator of the ripening process of mango in association with the degradation of chlorophyll, coupled with the loss of greenness in fruit [48]. The value of a* was the largest in ‘nam dok mai’ (6.74) and the lowest in ‘mahachanok’ (3.28). The increase in yellowness as depicted by b* values is attributed to an increase in carotenoid content [49]. The yellowness of mango was the greatest in ‘chok anan’ (43.09) and the least in ‘nam dok mai’ (36.63). Color change in ripe fruit is caused by a reduction in chlorophyll, leading to the synthesis of different types of anthocyanins within the vacuoles [50]. Along with that, carotenoids such as β-carotene, xanthophyll esters, xanthophylls, and lycopene accumulate during the process of ripening in the plastids [51].

The average values of geometric mean diameter (D_g_) ranged from 78.66–85.50 with the highest and the lowest values being that of ‘mahachanok’ and ‘kaew’, respectively. The geometric mean diameter represents the central tendency of the primary dimension and is normally used as parameter when designing fruit sorting machines [52,53]. Our result showed that the D_g_ values were greater than reported by Osadare et al. [54]. The arithmetic mean diameter (D_a_) is also an important indicator in determining the quality attribute of a particular mango variety according to size and maturity stage [55]. The result showed that size of the ripe mangoes of different varieties varied between 81.76 and 96.30 mm. Both D_g_ and D_a_ are also regarded as physical parameters for fruit grading [56]. The aspect ratio (R_a_) is related to the width-to-length ratio determination, which indicates an ellipsoid shape during the process of fruit development [57]. The higher value in ‘chok anan’ (66.98) and ‘kaew’ (63.32) denotes that the shapes of these mango varieties were round, similar to ‘cogshall’ mango which is triaxial ellipsoid in shape [57]. The sphericity that provides an indication of the tendency of shape varied from 258.13–297.95. The high sphericity of ‘chok anan’ showed its tendency of the morphology toward a sphere. The lowest aspect ratio and sphericity in ‘mahachanok’ indicated its inclination toward an elongated oblong shape. It is worth highlighting that both parameters intercorrelate with each other, and greater values of R_a_ and sphericity denote more advanced ripening stages of the fruits [58]. Additionally, a higher fruit ripeness leads to a greater content of pectin from fruit peel [59].

The surface areas (S) of mango fruits varied from 180.13–213.31 cm^2^. The highest area was obtained from ‘mahachanok’ (213.31 cm^2^), followed by ‘nam dok mai’ (208.09 cm^2^), ‘chok anan’ (188.70 cm^2^), and ‘kaew’ (180.13 cm^2^). Meanwhile, ‘mahachanok’ also illustrated the highest ratio of peel to total fruit weight (16.64%), whereas ‘chok anan’ and ‘nam dok mai’ had the lowest ratio at approximately 14%. Both surface area and peel-to-fruit ratio may be associated with pectin content due to the naturally heterogeneous polysaccharide present in plant cell walls, especially between the middle lamella [60]. However, the composition of pectin in plant cell walls varies as a function of the type and the variety [61]. In term of processing, ‘nam dok mai’ gave the largest yield of flesh (73%) and the least biomass (~26%), followed by ‘kaew’, while ‘mahachanok’ and ‘chok anan’ were much less economical for processing. The most frequently used varieties for processing are ‘sampee’ and ‘kaew’ for candies, ‘mahachanok’ for juice, and ‘chok anan’ for rehydrated mango products, in which heat treatment is commonly incorporated [12]. In comparison with yield compositions in other varieties, Abdualrahm [62] and Anila and Radha [63] reported that peel, seed, and flesh weights of different mango varieties in India were in the ranges of 10–22%, 7–20%, and 58–81%, respectively. On the other hand, nine Hispanic mango varieties consisted of 6–12% peel weight, 4–12% seed weight, and 75–86% flesh weight [64]. We were particularly interested in the valorization of mango peel, and it seems that ‘mahachanok’ is a preferred variety with the highest peel-to-fruit ratio and total biomass. A similar study of Sommano et al. [11] also found that ‘mahachanok’ gave the maximum yield of 6.0% of peel-to-fruit ratio, and a substantial amount of pectin could be obtained.

**Figure 1:**
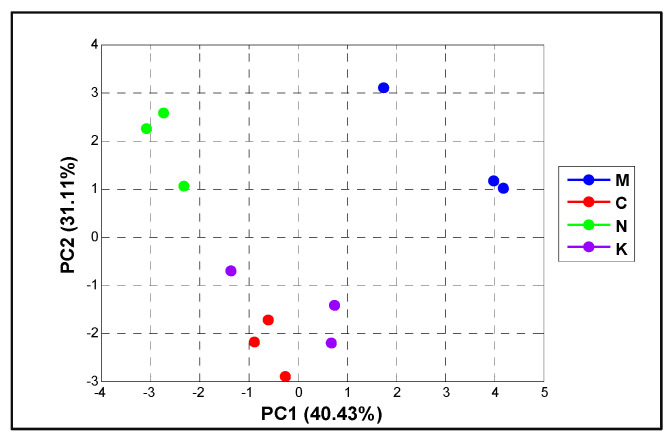
The chemometric PCA score plot based on the physiological characteristics and mango varieties (‘mahachanok’; M, ‘chok anan’; C, ‘nam dok mai’; N, ‘kaew’; K).

Additionally, to analyze the overall influence of fruit physical properties and mango varieties, we used a chemometric PCA as presented in Figure 1. All physical properties were combined to reduce the size of the analyzed samples. The first two dimensions of the PCA accounted for a total of 71.54% across the PCA score plot (PC1 = 40.43% and PC2 = 31.11% of the variance). The PCA plot illustrated that ‘chok anan’ and ‘kaew’ were somewhat identical in terms of their physiology, whereas ‘nam dok mai’ and ‘mahachanok’ were highly distinctive from others.

### 2.2. Physicochemical Characteristics of Mango Peel

#### 2.2.1. FT-IR

**Figure 2:**
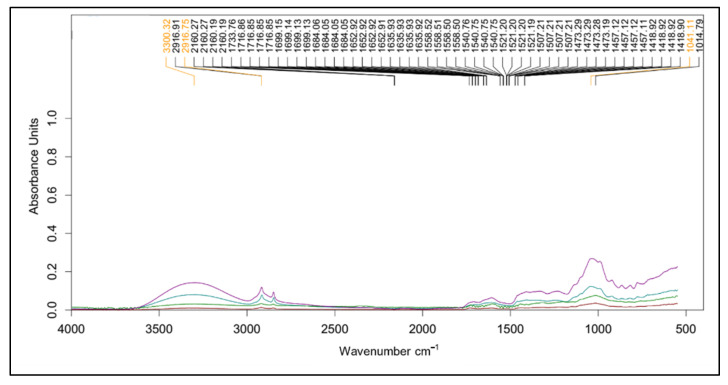
The FT-IR spectra of mango peels var. ‘mahachanok’ (**―**), ‘chok anan’ (**―**), ‘nam dok mai’ (**―**), and ‘kaew’ (**―**) from 600 to 4000 cm^−1^ (*x*-axis) in terms of absorbance units (*y*-axis).

FT-IR was used to identify the functional groups of the phytochemical compositions of mango peel (Figure 2). The FT-IR region ranged from 600 to 4000 cm^−1^. This demonstrates the similarity of the absorbance patterns in peel from different varieties. The broad and intense peak at around 3400 cm^−1^ represents stretching of O–H group due to inter- and intramolecular hydrogen bonding of polymeric compounds such as alcohols, phenols, and carboxylic acids, as in pectin, cellulose, and lignin [65]. The small peak at 2900 cm^−1^ indicates C–H stretching of the CH_2_ groups [66,67]. The small absorption at around 1730 cm^−1^ shows the characteristic of esterified pectin, arising from the ester carbonyl stretching band [68]. The region at wavenumbers between 1500 and 1800 cm^−1^ is associated with the assessment of the degree of methylation [69]. The region between 900 and 1200 cm^−1^ is accordingly referred to as the ‘fingerprint’ for carbohydrates, especially in terms of sugar composition [70]. The peaks relate to the characteristics of pectin polysaccharides (polygalacturonic acid) identified at 962, 1024, 1099, 1156, and 1223 cm^−1^, which were assigned to C–O bending, C–C stretching, C–O stretching, C–H stretching, and C–O stretching, respectively [71]. The peaks at 1370 cm^−1^ could be the symmetric stretching of –COO− of pectin [72]. The FT-IR patterns consequently verify that the mango peel was composed of pectin.

#### 2.2.2. Scanning Electron Microscopy (SEM) and Light Microscopy (LM)

**Figure 3:**
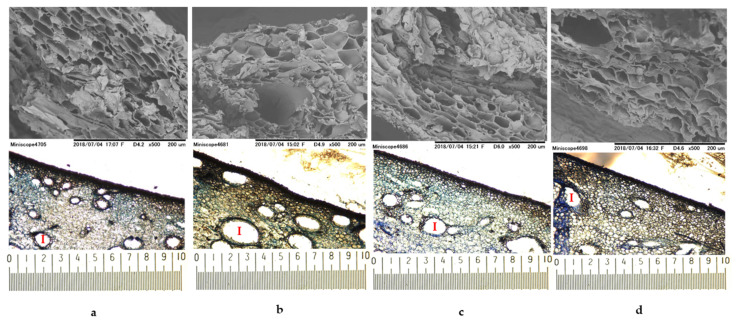
The SEM and LM images of mango peels var. ‘mahachanok’ (**a**), ‘chok anan’ (**b**), ‘nam dok mai’ (**c**), and ‘kaew’ (**d**). The images were viewed at ×500 and ×50 (0.1 mm/div).

The mango peel structure was characterized using SEM and LM as illustrated in Figure 3. SEM images showed that cell packings of ‘mahachanok’ (a) and ‘nam dok mai’ (c) were similar, and cells had an angular polyhedral shape with a flat cell compartment, as well as great intercellular space. On the other hand, ‘chok anan’ (b) cells were spherical, with a large compartment and less intercellular space. The cellular profile of ‘kaew’(d) was slightly irregular, with a flat compartment and no obvious intercellular space. According to LM observations, the intercellular space characteristics (I) of all mango varieties were associated with the SEM visualization. The variety of ‘chok anan’ exhibited a larger size and more space, while ‘kaew’ showed a small space and fewer cells. In addition, the color of cell components stained with toluidine blue O could define the different chemical compositions of each peel variety. Toluidine blue O is a cationic dye that binds to negatively charged groups and provides different colors, including a pinkish purple color when reacting with carboxylated polysaccharides such as pectin, a green, greenish blue, or bright blue color when reacting with aromatic substances such as lignin and tannins, and a purplish or greenish blue color when reacting with nucleic acids [73,74]. From the LM results, it could be implied that all varieties were composed of pectin, lignin, or tannin and nucleic acids because of their cell color. However, ‘chok anan’ and ‘kaew’ presented a high intensity of pinkish purple color as compared to the others. Therefore, they possibly contained a high content of pectin.

To describe and compare the mango peel anatomy of each variety, we then quantified cell structure compositions, as shown in Table 2. The greatest value of epidermis thickness was obtained in ‘chok anan’, followed by ‘mahachanok’, ‘nam dok mai’, ‘kaew’. While ‘mahachanok’ showed the highest cell density and number of intercellular spaces, the lowest of these values were seen in ‘kaew’. However, ‘mahachanok’ gave the lowest size of intercellular space. The size of the cell compartment of ‘chok anan’ and ‘nam dok mai’ was somewhat similar, whereas ‘mahachanok’ had the smallest. The decrease in the number of cell layers was associated with peel firmness, which declined along with the advancement in the ripening stage [75]. During ripening in most fruits, parenchyma cell walls are considerably modified, altering their mechanical properties, and the walls of some cells collapse while some cells fuse with others [76]. Cell-wall and middle lamella modifications (dissolution and depolymerization of pectin, hemicellulose, and cellulose) leading to fruit softening result from the action of cell-wall-modifying enzymes, including polygalacturonase, pectin methylesterase, pectate lyase, β-galactosidase, and cellulase [77,78,79]. Accordingly, the differences in anatomical components in each mango peel variety were possibly due to the variation of maturity stage. Similar results were also found in the peel of ‘hom thong’ banana at different maturation stages [75]. Noteworthily, soluble pectin is a general indicator of fruit ripening. To add to this point, the ripening process provides an increase in the content of pectin loosely bound to the cell wall [80,81], which occurs in parallel with a decrease in the amount of covalently bound pectin [82,83,84]. This implies that the ripening stage and the extraction ability of pectin from the cell are intercorrelated.

#### 2.2.3. Proximate and Sugar Compositions

The proximate analysis and sugar types of dried mango peels are shown in Table 3. Peels of all mango varieties contained ~59–69% moisture content, >9% carbohydrate, moderate contents of crude fiber, crude protein, and crude fat, and a low content of ash. The moisture content of fruit peel is an indicator of fruit ripeness, and ripe fruit typically consists of higher moisture content than raw fruit [85]. The moisture content values in previous reports were higher than those observed in this study, which might be due to the different ripening stages [86,87].

Carbohydrate contents of all samples were slightly variable. The contents of ‘nam dok mai’, ‘chok anan’, ‘mahachanok’, and ‘kaew’ were 11.45%, 11.23%, 10.53%, and 8.93%, respectively. Carbohydrate was the most abundant macronutrient in mango peel. This was in conformity with other studies (15–30%) [62,63,64]. Major carbohydrate compositions in ripened mango fruit are sugars (glucose, fructose, and sucrose) and others such as starch and pectin [88]. Pectin is a structural carbohydrate abundant in mango fruit and is considered as an important gelling sugar. When fruit is unripe, pectin is accumulated, whereas, during ripening, the pectin molecular weight decreases [88,89]. This is attributed to the hydrolysis activity of pectin enzymes at this stage [90].

There was a significant difference in crude protein content in peels of all varieties, ranging from 7.03–8.06%. The highest yield was seen in ‘kaew’, while ‘nam dok mai’ gave the lowest yield. When compared with other studies, the protein content in our research was much higher [62,63,91]. The content of protein in the peel may be correlated with pectin modification during the maturity stage [84]. The reason is that pectin is naturally solubilized and sequentially disassembled because of the loss of neutral sugars from the side-chain via depolymerization during the ripening stage [92,93,94,95]. The incidence involves pectolytic enzymes such as polygalacturonase, pectin methylesterase, and galactosidase. As a consequence, the pectin molecular weight decreases, which is in line with the concomitant loss of neutral sugars (arabinose and galactose), associated with the softening of mango [96,97,98]. Nevertheless, the extension of these changes varies greatly among different species [80,81].

Crude fat contents of ‘mahachanok’, ‘nam dok mai’, ‘kaew’, and ‘chok anan’ were 2.48%, 1.86%, 1.68%, and 1.51%, respectively. The content of crude fat was rather low when compared to other components. Studies on different varieties of mango peel reported values of fat content between 4% and 5% [99,100]. The fat content in mango peel was analyzed and reported in the form of fatty acid by Maldonado-Celis et al. [98] and Saleem Dar et al. [89]. They observed that the fatty-acid content increased during the ripening stage. Bandyopadhyay and Gholap [101] also found that the ratio of palmitic–palmitoleic acid in ripe mango could be applied as an index of aroma and flavor of mangoes. The contents of crude fiber in ‘kaew’, ‘nam dok mai’, ‘mahachanok’, and ‘chok anan’ were 20.53%, 19.90%, 12.44%, and 10.92%, respectively. It is worth noting that the contents of crude fiber in var. ‘kaew’ and ‘nam dok mai’ accounted for about one-fifth of the total dried sample weight. Therefore, both varieties could be used as an ingredient in food products with supplemented dietary fiber in order to achieve higher profitable utilization. Nonetheless, the fiber contents of mango peel in this study were greater than the quantity reported in ‘amarpali’ (8.4%) and ‘dasheri’ (6.7%) by Tokas et al. [87], as well as in ‘nyala’ (4.5%), ‘edelfursan’ (4.2%), and ‘kaboom’ (4.4%) by Abdualrahm [62]. The ash content of the mango variety ‘mahachanok’ was greatest (0.54%), while others were not statistically different (0.24–0.27%). Ash consists of the important nutritional ingredients, especially minerals, as well as both micro and macronutrients, which are very important for the normal physiological functions of the human system [102].

The major sugar compositions of all mango peels were fructose and xylose. The contents of both sugars in each variety were not apparently distinct. Meanwhile, glucose and sucrose were not detected (Table 3). Fructose is the main monosaccharide during the pre-climacteric phase, while xylose, derived from hemicellulose, is the second most common sugar in nature and accounts for 18–30% of lignocellulose hydrolysate sugars [103]. It comes as no surprise that we detected a large quantity of xylose from peel byproduct. In general, mango flesh is predominantly composed of sucrose, fructose, and glucose in the order of highest to lowest content [88]. Kumar et al. [104] also found that the extracted sugars obtained from mango peel were mostly glucose, sucrose, and fructose. Nevertheless, the sugar types in mango peels are probably correlated with the neutral sugars attached on the side-chain of the pectin structure [96,97,98].

**Figure 4:**
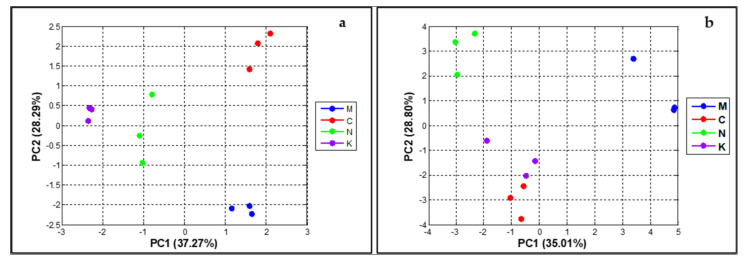
The chemometric PCA score plots of proximate compositions (**a**) and of physiological characteristics and proximate compositions (**b**) (‘mahachanok’; M, ‘chok anan’; C, ‘nam dok mai’; N, ‘kaew’; K). The representative points of each variety that are far apart indicate that the characteristics of fruit and peel of the mangoes are significantly different.

The relationship of proximate compositions of peel and the mango varieties was determined using PCA, whereby the first two dimensions of the PCA accounted for a total of 65.56% of variance across the PCA score plot (PC1 = 37.27% and PC2 = 28.29% of the variance). As presented in Figure 4a, the four varieties could be evidently classified on the basis of their chemical components since the score values of each variety were significantly different. Accordingly, the chemometric PCA of the phytochemical was appropriate for variety classification of the mango.

We were also interested in the relationship of the combinations of fruit physiology and peel characteristics and the mango varieties (Figure 4b). The first two dimensions of the PCA described a total of 63.81% of the variance across the PCA score plot (PC1 = 35.01% and PC2 = 28.80% of the variance). The PCA pattern was greatly analogous to Figure 1, describing that ‘chok anan’ and ‘kaew’ could not be remarkably separated because of the slight difference in their score values, whereas ‘nam dok mai’ and ‘mahachanok’ were clearly clustered from other varieties. From these results, it can be assumed that chemical properties hold greater potential for the categorization of mango varieties when compared with physiological characteristics. Therefore, the different varieties of mango were composed of distinctive proximate compositions in their peels.

### 2.3. Chemical Characteristics of Mango Peel Pectin

The Eq.W is an index of free galacturonic acid content in the pectin. Absolute pectic acid is composed entirely of polygalacturonic acid [105]. without any methyl ester groups [106]. The Eq.W of pectin from these mango varieties could be categorized into two levels. The highest level was 1000–2000 mg/mol from peels of ‘mahachanok’, ‘chok anan’, and ‘kaew’, while the peel of ‘nam dok mai’ showed the lowest level at about 600 mg/mol (Table 4). The values are comparable with citrus pectin, which illustrated ranges of Eq.W between 635.63 and 2219.39 mg/mol depending on the extraction method [71]. The larger Eq.W could be due to higher partial degradation of pectin side-chain leading to pectin purification and free acid being obtained [106,107]. The partial degradation of pectin is probably due to pectolytic enzymes (polygalacturonase, pectin methylesterase and galactosidase), leading to a decrease in pectin molecular weight with attendant loss of neutral sugars, together correlated with more ripeness in several mango varieties [96,97,108]. Subsequently, it is possible that a greater ripeness of mango fruit leads to higher values of Eq.W.

Meanwhile, the Mox could be categorized into 3 levels: the high level (Mox 20.0–40.0%) including ‘mahachanok’ and ‘keaw’, the moderate level (Mox 10.0–20.0%) including ‘nam dok mai’, and the low level (Mox <10.0%) including ‘chok anan’. Mox content is an essential indicator of pectin setting time, related to its distribution ability in water and gel formation ability [109,110,111]. Commercially, a high-Mox pectin (generally at 8–11% Mox) can form gels at a high sugar content (>65% sugar), while a low-methoxyl pectin (LMP) with less than 7% Mox can form gels at a lower sugar content [112]. Depending on the DE, pectin can be divided into two groups: pectin with DE higher than 50%, known as high-methoxyl pectin (HMP), and DE lower than 50%, known as low-methoxyl pectin [113]. The DE of extracted pectin from various mango varieties ranged between 56.88% and 92.93%, indicating that all mango peel pectin was of HMP type. Although, the pectin obtained from mango peel var. ‘chok anan’ was composed of a DE content higher than 50%, the Mox value was fairly low (3.99%). Therefore, the pectin extracted from ‘chok anan’ could be classified as an LMP, which can be used to supplement a low-sugar diet.

### 2.4. Chemometric Studies of Fruit Physiological and Peel Proximate Compositions with Pectin Qualities

**Figure 5:**
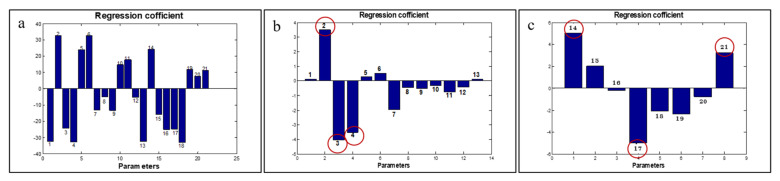
The corresponding PLS values of the impact of physiological and proximate characteristics on DE (**a**); the corresponding PLS values of the impact of physiological properties on DE (**b**); the corresponding PLS values of the impact of proximate compositions on DE (**c**). Physiological properties of mango fruit were %peel (1), %flesh (2), %seed (3), %total biomass (4), L* (5), a* (6), b*(7), D_g_ (8), D_a_ (9), R_a_ (10), Φ (11), surface area (12), and peel-to-fruit ratio (13); the proximate compositions were crude fiber (14), crude protein (15), crude lipid (16), moisture content (17), ash (18), carbohydrate (19), fructose (20), and xylose (21). The bar graphs of each parameter indicate positive and negative impacts on DE value.

To examine the relationship of fruit physiological and peel physicochemical properties with the qualities of pectin, PLS models were established. It should be noted here that the data were standardized prior to the PLS modeling to ensure that each parameter equally influenced the estimation of the models. The correlation graphs between the observed parameters and the predicted pectin quality are presented in Table S1 (Supplementary Materials). The values of *R*^2^, *Q*^2^, and their standard errors are summarized in Table 5. The PLS models were of inordinate predictive performance (*R*^2^ > 0.7), while the *Q*^2^ values of the trained samples were low in all cases. Tandee et al. [114] described that predictive errors using the test sampling mode should be slightly greater than those of the auto-predictive mode, which is in line with our analysis. As shown in Table 5, the relationship between the physiological properties and Eq.W depicted high *Q*^2^ scores, while the others performed poorly. The relationship between the proximate properties and the DE value showed the lowest score of *Q*^2^, whereas the combined properties with all parameters were considerably acceptable with the exception of the Mox value. This could be due to the variation of the mango varieties (Figure 4b); however, our study did not determine the influence of the variety variation. Looking at the predictive models based on the highest *Q^2^* scores presented, we were able to pick up a strong relationship of the physiological and phytochemical properties with the DE value. To comprehend the influence of the analyzed parameters in each model of interest; we used PLS regression coefficients and selected the top three parameters illustrating the highest coefficient values (Figure 5).

Based on the coefficient values, the biomass yield parameters such as flesh (No. 2), seed (No. 3), total biomass (No. 4) (Figure 5b), crude fiber (No. 14), moisture content (No. 17), and xylose (No. 21) among the proximate properties (Figure 5c) had a strong influence on the model prediction of DE. It could be described that a higher flesh yield of mango fruit led to a greater DE value of the extracted pectin. On the other hand, fruit with high percentage of seed and total biomass had a tendency to give pectin of low-methoxyl type. Fruit biomass is an indicator used to determine the ripening stage of fruit. Peter et al. [115] reported that the ripening stages of ‘dodo’ mango fruit had a positive correlation to the flesh yield, whereas the seed and peel volumes were slightly changed. Therefore, it is likely that the quality of the extractable pectin depends upon fruit maturity. According to the phytochemical properties, the contents of crude fiber and xylose in mango peel resulted in a larger value of DE, whereas moisture content had the inverse effect. Mostly during ripening, the decrease in moisture content of mango peel happens during the diffusion of moisture from the flesh to the peel, along with carbohydrate hydrolyzation and alteration of crude fiber. These incidents are associated with an increase in the amount of the soluble pectin [115,116,117].

## 3. Materials and Methods

### 3.1. Physical Characteristics of Mango Fruit Varieties

#### 3.1.1. Collection of Mango Samples

Four mangoes varieties (‘mahachanok’, ‘chok anan’, ‘nam dok mai’, and ‘kaew’) were harvested at a commercial ripening stage with their specific gravities in the range of 1.01–1.02, as described by Wongkaew et al. [6]. The mangoes were obtained from the orchard of Maejo University located in Sansai district, Chiang Mai, Thailand.

#### 3.1.2. CIE Color Spacing

The color measurement was repeated six times at different positions over the fruit surface using a handheld color spectrophotometer (NS800, 3nh, China). Before each measurement, the instrument was calibrated using a white ceramic tile. The measurement was assessed using the CIE Lab system, where L* denotes lightness on a 0–100 scale from black to white, a∗ denotes (+) red or (−) green, and b∗ denotes (+) yellow or (−) blue.

#### 3.1.3. Physical Properties

Linear dimensions, including length (L), width (W), and thickness (T), were measured using a digital vernier caliper with an accuracy of 0.01 mm. The physical properties were calculated according to the following equations [118, 119, 120]:

(1) $$\mathrm{Arithmetic}\;\mathrm{mean}\;\mathrm{diameter}\;(D_{a}):\;D_{a}=\frac{\left(L+W+T\right)}{3},$$

(2) $$\mathrm{Geometric}\;\mathrm{mean}\;\mathrm{diameter}\;(D_{g}):\;D_{g}{=(\mathrm{LWT})}^{1/3},$$

(3) $$\mathrm{Aspect}\;\mathrm{ratio}\;(R_{a}):\;R_{a}=\left(\frac{W}{L}\right)\;\times \;100,$$

(4) $$\mathrm{Sphericity}\;(\Phi ):\;\Phi =\left(\frac{{\mathrm{LWT}}^{1/3}}{L}\right),$$

(5) $$\mathrm{Surface}\;\mathrm{area}\;(S):\;S=\frac{{\pi \mathrm{BL}}^{2}}{\left(2L\;-\;B\right)}\;;\;\mathrm{where}\;{B=(\mathrm{WT})}^{0.5},\;{S=\pi (D}_{g}{)}^{2}.$$

### 3.2. Physicochemical Characteristics of Mango Peel

#### 3.2.1. Preparation of Mango Peel Powder

Peel was removed from the ripe mangoes prior to cutting into small pieces, washing with tap water, blanching with hot water at 95 °C for 10 min, draining, and leaving to cool at room temperature. It was then dried at 60 ± 1 °C until a moisture content of 4–6% was reached [10,121]. The dried peel was ground to fine powder using the high-speed mode of a food processor and passed through a sieve, resulting in a final mass of particles smaller than 0.6 mm in diameter [122,123].

#### 3.2.2. Fourier-Transform Infrared Spectrophotometry (FT-IR)

The FT-IR spectra were acquired using a compact infrared spectrometer (Alpha II Bruker, Bruker Corporation, Billerica, MA, USA) equipped with a deuterated triglycine sulfate (DTGS) detector. Each powder sample was scanned by placing the sample on the platinum ATR with a durable magnetic diamond interface. The spectrum was verified in transparent mode from 500 to 4000 cm^−1^, with a resolution of 4.0 cm^−1^ [12]. Each IR spectrum was validated with reference standards.

#### 3.2.3. Scanning Electron Microscopy (SEM)

Fresh mango peel was cut into 1 × 1 × 0.2 cm pieces and fixed with a mixed solution of formaldehyde and glacial acetic acid in a ratio of 1:1 at a temperature of 4 °C for 12 h. Subsequently, the fragments were dehydrated in an ethanol series and dried using a freeze-dryer. Mango peel was attached onto a specimen stub with a double-sided tape and sputter-coated with platinum [12,66]. The images were viewed at magnifications of ×500 using SEM (JELO JSM-5910, JEOL Ltd., Japan) with an accelerating voltage of 10 kV.

#### 3.2.4. Light Microscopy (LM)

Similarly sized mango peels were fixed and dehydrated according to the protocol of SEM preparation. Afterward, the materials were fixed and embedded in paraffin at 60 °C for 12 h. Sections (about 1 mm thick) were cut with a ultramicrotome and fixed to microscope slides. Sections were stained with toluidine blue O solution in 0.1 M phosphate buffer (pH 6.8). The samples were observed using an inverted light microscope according to the modified method of Rongkaumpan et al. [124].

#### 3.2.5. Proximate Compositions

Air-dried mango peel samples were used for proximate analyses with the exception of the moisture content, which was analyzed from fresh mango peels. The proximate composition analyses were carried out according to the methods of Association of Official Analytical Chemists (2000) [125]. Total carbohydrate contents were calculated using the following equation:

% Carbohydrate = 100 − (% moisture content + % crude protein + % ash + % crude fat + % crude fiber).(6)

#### 3.2.6. Sugar Compositions

Two grams of the peel powder samples were extracted with 20 mL of 80% methanol for 30 min in a shaker at room temperature. The extracts were filtered through filter paper (Whatman No. 1), and the residue was re-extracted under the same condition. The combined filtrate was evaporated in a rotary evaporator at a temperature below 45 °C. The extracts obtained after evaporation of methanol were used for the analyses of sugar content via HPLC. The mixture was separated in Shimadzu^®^ Prominence™ LC-20A System, Japan, with a reversed-phase HPLC column on RezexTM RHM Monosaccharide H+ (8%) (Phenomenex Inc., Torrance, CA, USA), LC column 300 × 7.8 mm column, using degassed water as mobile phase at flow rate of 0.6 mL/min. Pure samples of d-(+)-arabinose, d-(+)-xylose, d-(+)-glucose, d-(+)-fructose, and d-(+)-sucrose were used as standards [126].

### 3.3. Chemical Characteristics of Mango Peel Pectin

#### 3.3.1. Extraction of Pectin from Mango Peel Using Microwave Technique

Twenty grams of mango peel powder was suspended in 600 mL of diluted acidic solution (distilled H_2_O adjusted to pH 1.5 with 2 M HCl) and soaked for 20 min at room temperature. The slurry was heated in a microwave oven (ME711K-XST, Samsung, Thailand) with an optimal output power (700 watts for 3 min) followed by cooling to room temperature [12]. The solution was filtered and pressed manually using a nylon cloth. The filtrates were centrifuged at 5000× *g* for 20 min to eliminate any remaining coarse particles. Pectin was precipitated from the supernatant by adding the same volumes of ethanol (95%), before being mixed and stored in a refrigerator at 4 °C for 30 min. The separation was achieved by vacuum filtration. The obtained pectin was dried in a hot-air oven at 40 °C until constant weight was reached [127]. The yield of pectin was calculated from the following equation [123]:

(7) $$\mathrm{Yield}\;\left(\%\right)=\left(\frac{M_{0}}{M}\right)\;\times \;100,$$

where M_0_ (g) is the weight of dried pectin, and M (g) is the weight of dried mango peel powder.

#### 3.3.2. Mango Peel Pectin Characterizations

##### Equivalent Weight (Eq.W)

The equivalent weight (Eq.W) was determined using the method of Sommano et al. [11]. Briefly, 0.5 g of dried pectin was dissolved in 100 mL of distilled water at 25 °C and stirred for 2 h until completely dissolved. One gram of sodium chloride was added and titrated with 0.1 M of sodium hydroxide (NaOH) using five drops of phenol red as an indicator. Eq.W was calculated using the following equation:

(8) $$\mathrm{Eq}.W=\frac{1000\;\times \;\mathrm{pectin}\;\mathrm{powder}\;(g)}{\mathrm{NaOH}\;\mathrm{concentration}\;\left(N\right)\;\times \;\mathrm{NaOH}\;\mathrm{volume}\;(\mathrm{mL})}.$$

##### Methoxyl Content (Mox) and Degree of Esterification (DE)

The methods of Pinheiro et al. [128] were followed. Dried pectin (0.2 g) was stirred in CO_2_-free distilled water (20 mL) until fully dissolved. One gram of NaCl was added to the solution, prior to titrating with 0.1 N NaOH in the presence of phenolphthalein. The volume was recorded as the initial titer (V_1_). Then, 0.1 N NaOH solution (10 mL) was added to a neutralized polygalacturonic acid sample after determination of the free carboxyl groups. The solution was mixed thoroughly until the color of the solution became purple. A few drops of the indicator (0.25 N HCl) were added, and the mixture was titrated with 0.1 N NaOH until the color turned from yellow to pink. The volume was noted as V_2_. Mox and DE were then calculated using the following equations:

(9) $$\mathrm{Mox}=\frac{{(N)(V}_{2})(E)}{1000\;(S)},$$

(10) $$\mathrm{DE}=\frac{V_{2}\;\times \;100}{V_{1}{+V}_{2}},$$

where S is the mass of dried pectin (g), Nis the NaOH concentration (N), V_1_ is the volume of NaOH used (mL), V_2_ is the volume of NaOH used (mL), and E is equivalent weight of methoxyl = 31.

### 3.4. Statistical Analysis

The analyses of physical and chemical properties in this experiment were carried out at least in biological and technical triplicates. Data was analyzed using one-way analysis of variance and Duncan’s test. Differences in values were considered significantly different when the *p*-value was <0.05. All statistical analysis was performed using IBM SPSS program v. 23.0 (Armonk, New York, NY, USA). Principal component analysis (PCA), partial least-squares regression (PLS), and PLS coefficient evaluations was conducted to comprehend the influence of mango varieties on the physiological and physicochemical characteristics using in-house MATLAB scripts (MATLAB V10.0, The Math Works Inc., Natick). Relationships between the parameters of interest and chemical qualities of pectin were fitted using PLS models, where fruit and peel characteristics were used as predictive parameters, while pectin qualities were used as responses. Standardization (STD) was used for data preprocessing to equalize the effect of each variable’s contribution to the model evaluation [129].

## 4. Conclusions

Chemometric analysis is able to elucidate the differences in mango varieties according to their physiological attributes and peel proximate compositions. In terms of MPP recovery, the percentages of flesh, peel, and total biomass, as well as contents of crude fiber, moisture, and xylose in the peels, can be used to justify the pectin type and its associated DE value. Future directions from our study can target the development of a nondestructive tool for biomass sourcing in the recovery process of high-quality pectin production.

## Figures and Tables

**Table 1:** Physical characteristics of different mango varieties.

Parameters	Mahachanok	Chok Anan	Nam Dok Mai	Kaew
	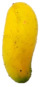	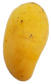	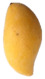	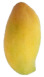
L*	68.83 ± 2.02 b	69.98 ± 2.72 b	72.26 ± 1.85 a	67.68 ± 3.07 b
a*	3.28 ± 2.68 b	5.55 ± 0.73 a	6.74 ± 0.86 a	3.41 ± 2.69 b
b*	40.66 ± 2.86 a b	43.09 ± 6.68 a	36.63 ± 1.48 b	39.70 ± 4.49 a b
**Dg (cm)**	85.50 ± 4.10 a	80.57 ± 2.86 b	84.61 ± 3.93 a	78.66 ± 3.80 b
**Da (cm)**	96.30 ± 5.96 a	83.33 ± 2.94 c	90.95 ± 4.11 b	81.76 ± 3.85 c
**Ra (%)**	40.79 ± 2.06 d	66.98 ± 3.05 a	50.44 ± 3.10 c	63.32 ± 6.27 b
**Φ** **(%)**	258.13 ± 19.16 b	297.95 ± 16.29 a	279.41 ± 22.26 a	280.02 ± 21.38 a
**S (cm^2^)**	213.31 ± 19.29 a	188.70 ± 13.40 c	208.09 ± 19.61 a	180.13 ± 16.78 c
**Peel-to-fruit ratio (%)**	16.64 ± 0.67 a	14.39 ± 0.57 c	14.42 ± 1.41 c	15.60 ± 0.66 b
**Flesh weight (%)**	66.69 ± 2.79 c	67.32 ± 2.63 c	73.15 ± 3.75 a	70.32 ± 1.88 b
**Peel weight (%)**	16.64 ± 0.67 a	14.39 ± 0.57 c	14.42 ± 1.41 c	15.60 ± 0.66 b
**Seed weight (%)**	16.66 ± 2.63 a	18.29 ± 2.37 a	12.43 ± 2.71 b	14.08 ± 2.02 b
**Total waste (%)**	33.31 ± 2.79 a	32.68 ± 2.63 a	26.85 ± 3.75 c	29.68 ± 1.88 b

Average ± standard deviation; different letters in each column denote a significant difference (*p* < 0.05).

**Table 2:** Anatomical components of mango peels from different varieties using SEM and LM.

Anatomical Components	Mango Varieties			
	Mahachanok	Chok Anan	Nam Dok Mai	Kaew
**1. Epidermis thickness**	+++	++++	++	++
**2. Cell density (0.01 mm^2^)**	++++	++	+++	+
**3. Number of intercellular space**	++++	+++	++	++
**4. Size of intercellular space**	+	++++	+++	++
**5. Cell compartment size (μm)**	200–400	400–500	300–400	400–500

Plus signs indicate the level from the highest (++++) to the lowest (+) of each anatomical component.

**Table 3:** Proximate and sugar analyses of different varieties of mango peels.

Mango Varieties	Proximate Composition (% *w*/*w*)						Sugar Types (% *w*/*w*)			
	Moisture in Fresh	Carbohydrate ^1,2,3^	Crude Protein ^1,2^	Crude Fat ^1,2^	Crude Fiber ^1,2^	Ash ^1,2^	Fructose ^1,2^	Xylose ^1,2^	Glucose	Sucrose
**Mahachanok**	66.51 ± 0.06 ^b^	10.53 ± 0.45 ^b^	7.50 ± 0.01 ^b^	2.48 ± 0.02 ^a^	12.44 ± 0.59 ^b^	0.54 ± 0.03 ^a^	31.23 ± 0.02 ^a^	29.88 ± 0.02 ^b^	n/d	n/d
**Chok anan**	68.88 ± 0.33 ^a^	11.23 ± 0.43 ^a b^	7.18 ± 0.02 ^c^	1.51 ± 0.02 ^d^	10.92 ± 0.37 ^c^	0.27 ± 0.01 ^b^	31.57 ± 0.03 ^a^	29.44 ± 0.06 ^b^	n/d	n/d
**Nam dok mai**	59.50 ± 0.06 ^d^	11.45 ± 0.28 ^a^	7.03 ± 0.41 ^d^	1.86 ± 0.02 ^b^	19.90 ± 0.28 ^a^	0.25 ± 0.04 ^b^	31.41 ± 0.07 ^a^	30.03 ± 0.03 ^a^	n/d	n/d
**Kaew**	60.54 ± 0.44 ^c^	8.93 ± 0.43 ^c^	8.06 ± 0.04 ^a^	1.68 ± 0.03 ^c^	20.53 ± 0.10 ^a^	0.24 ± 0.03 ^b^	31.35 ± 0.03 ^a^	29.73 ± 0.05 ^b^	n/d	n/d

^1^ Values are on a dry weight basis (d.w.). n/d: not detectable. ^2^ Average ± standard deviation; different letters in each row denote a significant difference (*p* < 0.05). ^3^ Calculated by difference with the other components of proximate content.

**Table 4:** Chemical characteristics of mango peel from different varieties.

Mango Varieties	Pectin Yield (%)	Eq.W (mg/mol)	Mox (%)	DE (%)
**Mahachanok**	13.67 ± 0.08 ^b^	1423.81 ± 41.24 ^a^	23.95 ± 16.55 ^a b^	89.85 ± 3.08 ^a^
**Chok anan**	15.07 ± 0.29 ^a^	1037.30 ± 4.96 ^b^	3.99 ± 0.02 ^b^	56.88 ± 0.78 ^c^
**Nam dok mai**	12.76 ± 0.71 ^b^	605.26 ± 9.12 ^c^	13.90 ± 2.57 ^b^	68.91 ± 6.38 ^b^
**Kaew**	7.65 ± 0.84 ^c^	1041.67 ± 38.19 ^b^	41.00 ± 14.74 ^a^	92.93 ± 1.76 ^a^

Data are expressed as mean ± standard deviation, *n* = 3. Eq.W = equivalent weight; Mox = methoxyl content; DE = degree of esterification. Average ± standard deviation; different letters in each row denote a significant difference (*p* < 0.05).

**Table 5:** *R^2^* and *Q^2^* values with their error scores obtained from the correlation graph of the expected and predicted pectin quality values with fruit physiological properties and nutritional compositions of peel using the PLS model.

Properties	Pectin Qualities	*R* ^2^	*Q* ^2^	RMSEC	RMSECV
**Physiological properties**	Eq.W	0.9782	0.4171	42.93	160.81
	Mox	0.6882	−0.2376	9.17	18.15
	DE	0.7682	−0.3614	7.32	22.57
	%Pectin	0.7823	−0.1145	1.33	3.08
**Proximate compositions of peel**	Eq.W	0.9841	0.5867	36.7	74.01
	Mox	0.9148	0.418	4.8	14.73
	DE	0.9617	−0.5432	2.98	4.18
	%Pectin	0.9849	0.7695	0.35	1.04
**Physiological and proximate compositions of peel**	Eq.W	0.9958	0.5534	18.84	116.28
	Mox	0.7456	−0.2376	8.29	18.26
	DE	0.9839	0.8323	1.93	5.26
	%Pectin	0.9826	0.4262	0.37	1.24

## Data Availability

Not applicable.

## Supplementary Materials

The following are available online at https://www.mdpi.com/article/10.3390/plants10061148/s1: Table S1: Correlation graph of PLS model.
