# Supplementary material for: Fruit Characteristics, Peel Nutritional Compositions, and Their Relationships with Mango Peel Pectin Quality
Source: Plants (Basel). 2021 Jun 4;10(6):1148. doi: 10.3390/plants10061148 (PMC8226707; doi:10.3390/plants10061148)
Supplement: Supplementary file 1 [file plants-10-01148-s001.zip › plants-1235704-supplementary.pdf]

Table S1. The correlation graph of the expected and predicted pectin quality values by fruit physiological properties and proximate compositions of peel using PLS model.

Properties	Pectin qualities			
	**Eq.W**	**Mox**	**DE**	**%Pectin**
Physiological properties	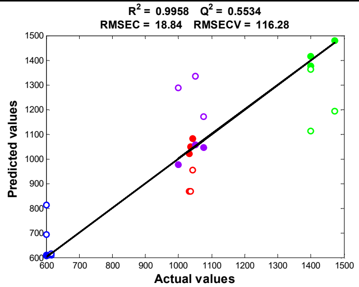	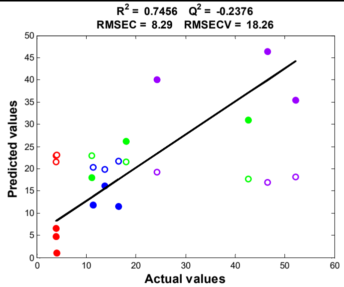	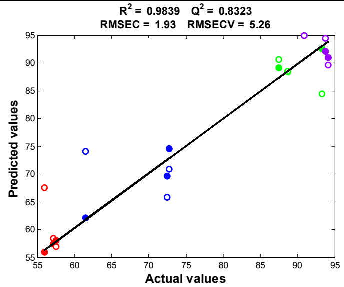	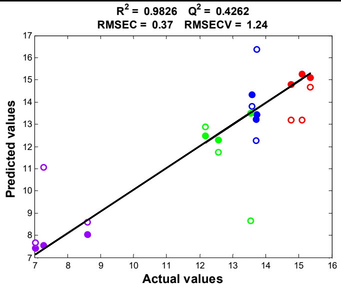
Proximate compositions of peel	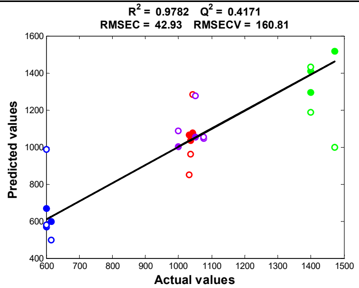	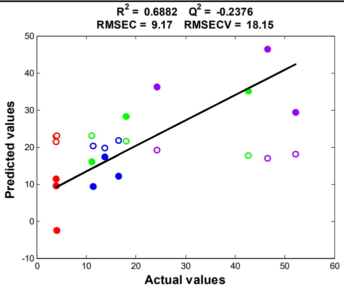	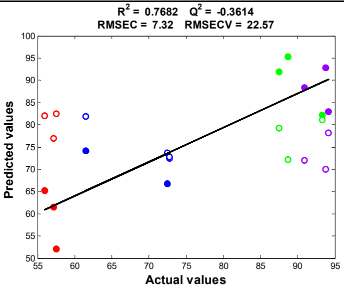	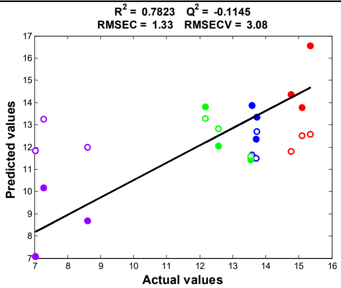
Physiological and Proximate compositions of peel	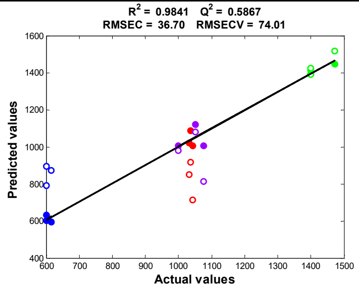	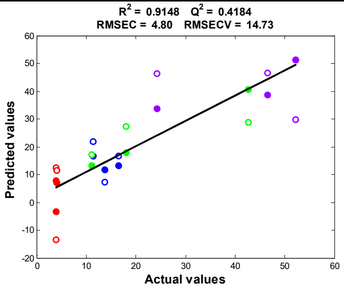	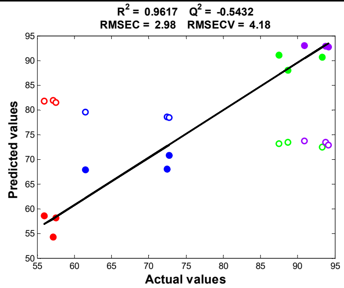	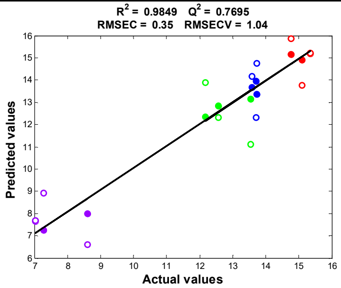
